# Effect of Combined Chitosan and Hyperbranched Poly-L-Lysine Based Coating on Prolonging the Shelf Life of Oyster Mushroom (*Pleurotus ostreatus*)

**DOI:** 10.3390/foods13010077

**Published:** 2023-12-25

**Authors:** Jianrui Sun, Ruirui Ren, Linlin Yao, Jinglan Li, Li Tong, Jiangfeng Yuan, Dahong Wang

**Affiliations:** Henan Engineering Research Center of Food Microbiology, College of Food and Bioengineering, Henan University of Science and Technology, Luoyang 471023, China; 15090320603@163.com (R.R.); linlinyao1121@163.com (L.Y.); lijinglan1207@163.com (J.L.); 15236675557@163.com (L.T.); jiangfengyuan@163.com (J.Y.); wangdahong2003@163.com (D.W.)

**Keywords:** oyster mushroom, chitosan, hyperbranched poly-L-lysine, preservation

## Abstract

To extend the shelf life of oyster mushroom (*Pleurotus ostreatus*), the effects of chitosan (CS) and hyperbranched poly-L-lysine (HBPL) combined treatment on quality characteristics, nutritional quality, storage characteristics, and enzyme activity of oyster mushroom during postharvest storage at 4 °C were investigated. The results showed that CS-HBPL combined treatment could significantly reduce rot degree and weight loss and significantly inhibit the browning of oyster mushroom. At the same time, the loss of reducing sugar, vitamin C, soluble protein, and total phenolic was significantly reduced. Compared with the control, CS-HBPL combined treatment could also significantly inhibit an increase in malondialdehyde (MDA) and significantly decrease the relative electrolyte leakage of oyster mushroom. In addition, the activities of catalase (CAT), superoxide dismutase (SOD), phenylalnine ammonialyase (PAL), and peroxidase (POD) were significantly improved, and the activity of polyphenol oxidase (PPO) was significantly inhibited in oyster mushroom. In conclusion, CS-HBPL combined treatment had a good protective effect on the membrane permeability damage of oyster mushroom and could effectively delay the oxidation of phenolic substances and browning of oyster mushroom. Therefore, CS-HBPL combined treatment can be used as a potential strategy to extend the storage time of oyster mushroom.

## 1. Introduction

Oyster mushroom, the fruiting body of *Pleurotus ostreatus*, is a popular edible fungus in China, which has a tender texture and delicious taste. It is rich in nutrients such as protein, polysaccharide, vitamins, and minerals [1], as well as bioactive substances such as lectins, phenols, and steroids. It has multiple health benefits, such as improving human metabolism, reducing cholesterol, and lowering blood pressure [2], and has gradually become the third most consumed edible fungi in the world after shiitake mushroom and bisporus mushroom. Fresh oyster mushroom contains over 90% moisture and is rich in nutrients with strong respiration. Moreover, the surface of oyster mushroom has no obvious protective structure, and the organic matter inside is easily decomposed and oxidized. Under normal temperature conditions, oyster mushroom shows characteristics of wilting, browning, and rot within 2–3 days [3], and the shelf life is very short, which seriously affects the edible value, commodity value, and appearance quality of oyster mushroom.

Coating preservation technology is a new technique proposed to lengthen the quality and shelf life of fruits and vegetables by delaying water loss, reducing respiration rate, preserving volatile compounds, and reducing microbial growth [4]. Chitosan (CS) is widely used in food additives because of its excellent biocompatibility, biodegradability, safety, film-forming ability, and antibacterial activity [5]. Chitosan has a broad-spectrum antibacterial effect, which can effectively inhibit food-borne pathogens, such as *Staphylococcus aureus*, *Salmonella enterica*, and *Escherichia coli*, and has the physical property of spontaneous film formation, which can form transparent and colorless films on the surface of food [6]. Currently, chitosan is extensively used in the preservation of fruits and vegetables, which can delay fruit senescence, inhibit bacteria, resist rot, ensure fruit and vegetable quality, and extend their storage life [7,8].

Different from the linear α-poly-L-lysine (PLL) and ε-poly-L-lysine (ε-PL) and dendritic polylysine, the hyperbranched poly-L-lysine (HBPL) has no regular structure, wide molecular weight distribution, highly branched structure, and abundant terminal amino groups. ε-PL is mainly obtained via bacterial fermentation and has been widely applied to the preservation of fruits and vegetables, meat products, and baked goods because of its broad-spectrum antibacterial activity [9,10,11]. The monomer of HBPL is L-lysine, an essential amino acid for humans. Therefore, the products of HBPL degradation can be considered non-toxic and have sufficient biological safety. Due to its highly branched structure and abundant terminal amino groups, HBPL is often used as a vector for gene delivery and drug delivery [12]. Scheper et al. [13] studied the possibility of HBPL as a drug delivery vehicle and proved that it did not cause cytotoxicity and had no adverse reactions in vivo. In another study, HBPL was modified to be coupled with folic acid and used as a contrast agent for magnetic resonance imaging (MRI), which further demonstrated its safety in vivo [14]. In addition, studies have begun to focus on the application of HBPL in food preservation [15]. The molecular structure of HBPL contains both ε-PL and PLL structural units. Previous studies have shown that HBPL has excellent antibacterial effects and good biosafety [13,16]. Therefore, HBPL can be used as a potential biological preservative for fruit and vegetable preservation.

At present, there are many research projects on the application of chitosan and ε-polylysine composite preservatives in food preservation. The composite film of ε-PL and chitosan was used to preserve citrus, and the tensile strength of the composite film decreased and the elongation at break increased with the rise of ε-PL content, and the composite film could effectively inhibit the decrease in the total soluble solids (TSSs) and vitamin C (Vc) content of citrus, thereby reducing the damage of citrus during packaging and storage [17]. The composite coating of chitosan and ε-PL could effectively prevent the bleaching of carrots [10]. The composite coating of chitosan, ε-PL, and ascorbic acid could effectively inhibit bacterial growth and increase pH value, total volatile basic nitrogen (TVB-N), and thiobarbituric acid-reactive substances in pork, while reducing the redness index of pork pieces [18].

The aim of this study was to investigate the effect of a combined CS and HBPL-based coating on improving the key quality characteristics of oyster mushroom during storage at 4 °C. In this study, the effects of CS and HBPL compound preservative on rot index, weight loss, browning degree, reducing sugar, Vc, total phenol, soluble protein, malondialdehyde (MDA), relative electrolyte leakage, catalase (CAT), superoxide dismutase (SOD), phenylalnine ammonialyase (PAL), peroxidase (POD), and polyphenol oxidase (PPO) activity of oyster mushroom during storage at 4 °C was studied to evaluate the application potential of CS and HBPL compound preservative in the preservation of mushroom and provide a new idea for extending the shelf life of oyster mushroom.

## 2. Materials and Methods

### 2.1. Materials

Hyperbranched poly-L-lysine (HBPL) was synthesized via a thermal initiation method [16]. A total of 27.45 g L-lysine hydrochloride was weighed and dissolved in a three-necked flask filled with 50 mL water. A total of 8.4 g KOH was weighed and dissolved in 30 mL water, which was added to a constant-pressure funnel and slowly dropped into the flask and neutralized for 2 h. The system was heated to 150 °C under nitrogen protection, without sealing the device to allow the water generated during the reaction to escape. After reaction for 24 h, 200 mL methanol was added to the system to dissolve the product. The KCl was removed via centrifugation, and the methanol was removed via rotary evaporation. An appropriate amount of water was added to dissolve it again, and dialysis with the interception of molecular weight 3500 dialysis bag for 3 days. The HBPL final product was obtained via freeze-drying.

Fresh and ripe oyster mushroom (*Pleurotus ostreatus*) was purchased from Dazhang Supermarket in Luoyang City, Henan Province, and immediately transported to the laboratory. The oyster mushrooms with complete shape, normal color, no decay, no damage, high moisture, and uniform size were selected (diameter of cap: about 5 cm; color of gills: white). Selected oyster mushrooms were randomly divided into four groups. Each group included 3 replicates, and each replication had 10 mushrooms.

### 2.2. Reagent

Chitosan and Folin-Ciocalteu’s phenol reagent were purchased from Shanghai Lanji Technology Development Co., Ltd., in Shanghai, China; anhydrous ethanol, trichloroacetic acid, hydrochloric acid, glacial acetic acid, and methyl alcohol were purchased from Tianjin Kemio Chemical Reagent Co., Ltd., in Tianjin, China; glucose, vitamin C, thiobarbituric acid, phenol, KH_2_PO_4_, Na_2_HPO_4_, NaOH, and Na_2_CO_3_ were purchased from Luoyang Haohua Chemical Reagent Co., Ltd., in Luoyang, China; 3,5-dinitrosalicylic acid, sodium tartrate, gallic acid, and natrium aceticum were purchased from Tianjin Deen Chemical Reagent Co., Ltd., in Tianjin, China; SOD, CAT, PAL, PPO, and POD kits were purchased from Beijing Solaybao Technology Co., Ltd., in Beijing, China.

### 2.3. Instruments and Equipment

The absorbance was measured using an ultraviolet–visible spectrophotometer (UV-1780, Shimadzu Corporation, in Kyoto, Japan). The mass was weighed using an electronic analytical balance (ME204E, Mettler Toledo Instrument Shanghai Co., Ltd., in Shanghai, China). The sample was centrifuged using a desktop-refrigerated centrifuge (H1850R, Hunan Xiangyi Instrument Development Co., Ltd., in Changsha, China). The electrical conductivity of the sample was determined using a table conductance meter (DDS-11C, Shanghai Precision Scientific Instrument Co., Ltd., in Shanghai, China).

### 2.4. Sample Treatment

#### 2.4.1. Preparation of Preservative

Chitosan solution (CS): a total of 7.5 g chitosan was accurately weighed, and 500 mL 1% glacial acetic acid was added and dissolved in a water bath at 75 °C. Hyperbranched poly-L-lysine solution (HBPL): a total of 582 μL 43% hyperbranched poly-L-lysine solution was filled to 500 mL. Chitosan and hyperbranched poly-L-lysine 1:1 complex solution (CS-HBPL): a total of 100 mL chitosan solution was evenly mixed with 100 mL hyperbranched poly-L-lysine solution. Blank control (CK): sterile water.

#### 2.4.2. Sample Treatment

The prepared HBPL, CS, CS-HBPL, and CK were evenly sprayed on the surface of each oyster mushroom with a sprayer 5 times. After treatment, the oyster mushroom was allowed to air-dry until no visible solution remained on the surface. The treated oyster mushroom was then packed into 18 cm × 20 cm bags of low-density polyethylene (PE) (0.04 mm thick). The PE gas transmission rates for O_2_, CO_2_, and H_2_O were 1078 × 10^−18^ mol m^−1^ s^−1^ Pa^−1^, 4134 × 10^−18^ mol m^−1^ s^−1^ Pa^−1^, and 2.8 × 10^−5^ to 6.5 × 10^−5^ g m^−2^ s^−1^, respectively.

The processed oyster mushroom was placed in a cool and dry place for natural air-drying. The dried oyster mushroom was placed in a perforated preservation bag, sealed, and labeled accordingly. Finally, the samples were moved to 4 °C, and the physical and chemical indexes were measured every 2 days until the 14th day.

### 2.5. Determination Indexes and Methods

#### 2.5.1. Rot Index

If shiitake mushroom did not rot, its rot index was 0; in the case of decay, the rot index was calculated according to Equation (1). According to the rotting area of the mushroom body, it was divided into two levels (level 1: rotting area < 50%; level 2: rotting area > 50%).

(1) $$Rot\;index=\frac{\sum \left(rot\;level\times number\;of\;oyster\;mushroom\;at\;this\;level\right)}{Total\;number\;of\;oyster\;mushroom\times highest\;level\;of\;decay}$$

#### 2.5.2. Browning Degree

A total of 10 g oyster mushroom was weighed and mixed with distilled water (1:10). After being homogenized at 4 °C for 2 min and standing for 30 s, the sample was centrifuged at 8000 r/min for 10 min (4 °C). The absorbance was measured at 410 nm. The browning level of oyster mushroom was indicated by 10 × A_410_ [5].

#### 2.5.3. Weight Loss

The weight loss rate of oyster mushroom was calculated using the weighing method according to Equation (2).

(2) $$Weight\;loss\;rate=\frac{\left(m_{1}-m_{2}\right)}{m_{1}}\times 100\%$$

where m_1_ was the initial mass of oyster mushroom and m_2_ was the mass of oyster mushroom in each period.

#### 2.5.4. Reducing Sugar Content

The method of DNS was used for determination. A total of 1 g of the cold mushroom cap was weighed, and 10 mL purified water was added to grind. Then, the grinding liquid was poured into a test tube and bathed at 80 °C for 30 min to leak out the reducing sugar. After cooling, the sample was centrifuged at 8000 r/min for 10 min at 4 °C. Then, 2 mL supernatant was absorbed, and 1.5 mL 3, 5-dinitrosalicylic acid was added, mixed thoroughly, and heated in a boiling water bath for 5 min. Then, the sample was metered volume with distilled water to 25 mL. The absorbance value was determined at 540 nm, and the content was calculated through the standard curve.

#### 2.5.5. Vitamin C Content

Ultraviolet spectrophotometry was used to determine the content of Vc [19], which was based on the absorption of Vc to ultraviolet and its instability to alkali. The difference in absorbance between the sample solution and the alkali-treated sample solution was determined at 243.4 nm, and the content of Vc could be calculated through the standard curve equation. A total of 10.0 g oyster mushroom was weighed into a mortar, and 10 mL of 1% HCl was added. The mixture was homogenized and transferred completely into a 50 mL volumetric flask and then filled up with purified water. An appropriate amount of diluted solution was transferred to the centrifuge tube and centrifuged at 8000 r/min for 10 min. The supernatant was collected as the oyster mushroom extract. Then, 1.0 mL of oyster mushroom extract was added to a 50 mL volumetric flask containing 2 mL 10% HCl, diluted with purified water, and shaken well, and its absorbance was measured. A total of 1.0 mL oyster mushroom extract, 10 mL distilled water, and 4 mL 1 mol/L NaOH solution were mixed well in a 50 mL volumetric flask, stood for 20 min, and then added to 4 mL 10% HCl; the absorbance was measured after mixing well. The difference between the extract and alkali-treated extract was the absorbance of the oyster mushroom sample. The concentration of vitamin C in the oyster mushroom sample could be calculated according to the standard curve equation. The content was calculated using Equation (3):

(3) $$Vitamin\;C\;content=\frac{c\times 100\times V_{2}\times V_{3}}{V_{1}\times 1000\times W}$$

where c was the vitamin C concentration calculated according to the standard curve equation; V_1_ was the volume of the sample solution absorbed when measuring absorbance; V_2_ was the total volume of the sample at constant volume; V_3_ was the total volume of the sample to be measured; and W was the quality of oyster mushroom.

#### 2.5.6. Total Phenols Content

A total of 2.0 g oyster mushroom cap was taken to grind in an ice bath with 10 mL of 95% ethanol, and then ultrasonic crushing was performed for 1 h. The supernatant was absorbed after centrifugation at 4 °C 8000 r/min for 10 min. Then, another 10 mL 95% ethanol was added to the remaining residue, and the above steps were repeated to mix the two supernatants. A total of 0.4 mL supernatant was transferred into a test tube, and then 2 mL Folin phenol reagent was added. After shaking the reaction mixture to avoid light for 5 min, 1.6 mL 7.5% Na_2_CO_3_ was added into the test tube and placed in the dark for 1 h. The absorbance was measured at 765 nm, and the total phenols content was calculated using the gallic acid standard curve. The content of total phenols was expressed as g gallic acid equivalent per kilogram (g GAE kg^−1^).

#### 2.5.7. Soluble Protein Content

The protein content of soluble protein was determined via the Coomassie brilliant blue method. A total of 2.0 g oyster mushroom was accurately weighed, and 6 mL 50 mM phosphate buffered (pH 7.0) was added. After being fully ground in the ice bath, the mixture was centrifuged at 10,000 r/min for 20 min, and the supernatant was collected for protein content determination. A total of 1 mL of the extract and 5.0 mL of Coomassie brilliant blue solution were mixed well. With the mixture standing at room temperature for 5 min, the absorbance was determined at 595 nm. The protein content was calculated using the standard curve drawn via bovine serum protein.

#### 2.5.8. Malondialdehyde (MDA)

The thiobarbituric acid method was used for the determination [5]. A total of 1.0 g oyster mushroom cap was accurately weighed and placed in a mortar to grind with 5 mL 10% trichloroacetic acid. The homogenate was centrifuged at 8000 r/min for 10 min. A total of 2 mL supernatant was transferred into a centrifuge tube, and then 2 mL 0.6% thiobarbituric acid and 2 mL distilled water were added. Incubating the reaction mixture in a boiling water bath for 15 min for color development, the mixture was cooled naturally. The mixture was centrifuged at 5000 r/min for 10 min, and then the supernatant was obtained. The absorbance was measured at 600, 532, and 450 nm, and the MDA content was calculated according to the following formula.

(4) $$C_{MDA}(µmol/L)=6.45(A_{532}-A_{600})-0.56A_{450}$$

The MDA concentration in the extract was calculated as follows:

(5) $$MDA\;concentration\;in\;the\;extract\;(µmol/mL)=\frac{C_{MDA}\times \frac{Reactive\;liquid\;volume\;(mL)}{1000}}{The\;amount\;of\;measured\;extraction\;liquid}$$

The MDA content in the sample was calculated according to the following formula:

(6) $$MDA\;content\;(µmol/g)=\frac{MDA\;concentration\;in\;the\;extract\times Total\;volume\;of\;extract}{Fresh\;weight\;of\;taken\;tissue}$$

#### 2.5.9. Electrolyte Leakage Rate

Perforating mushroom tissues at different storage stages with 0.5 cm × 0.5 cm punching, the tissues were washed three times with deionized water to separate the electrolyte. Then, the tissues were dried on filter paper and suspended in 40 mL deionized water. A magnetic stirrer was used to rotate the samples at 25 °C for 30 min, and the electrical conductivity was immediately recorded as P_0_. After 10 min, the electrical conductivity was again recorded as P_1_. The final electrical conductivity was recorded after the sample was boiled for 10 min and cooled to room temperature as P_2_ [20]. The relative conductivity was (P_1_ − P_0_)/(P_2_ − P_0_), which indicated the electrolyte leakage rate.

#### 2.5.10. Enzyme Activity Assay

Catalase (CAT), superoxide dismutase (SOD), peroxidase (POD), phenylalnine ammonialyase (PAL), and polyphenol oxidase (PPO) activity assay kits were used to determine CAT, SOD, POD, PAL, and PPO activity according to the manufacturer’s instructions.

### 2.6. Statistical Analysis

The main experiments were performed and repeated at least three times. The experimental results were presented as the mean ± SD (standard deviation). Statistical analysis was analyzed using SPSS 18.0, and *p* < 0.05 was taken as statistically significant.

## 3. Results and Discussion

### 3.1. Effects of CS and HBPL Treatment on Quality Characteristics during Oyster Mushroom Storage

**Figure 1:**
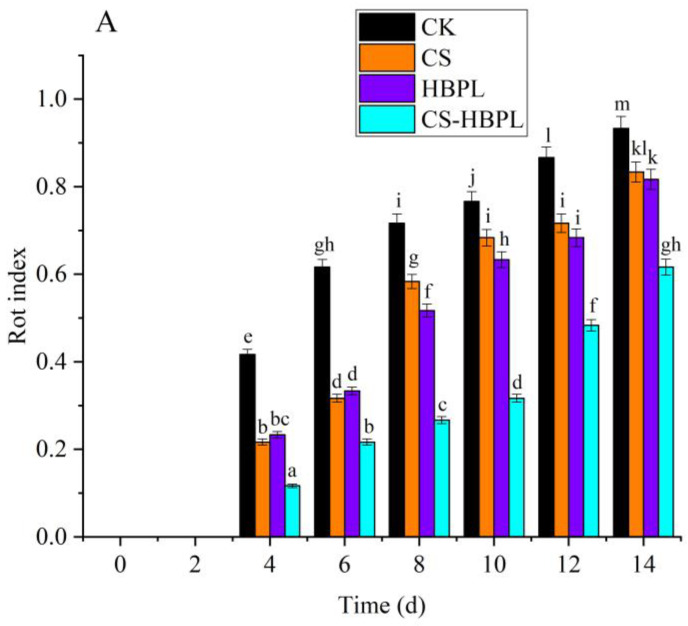

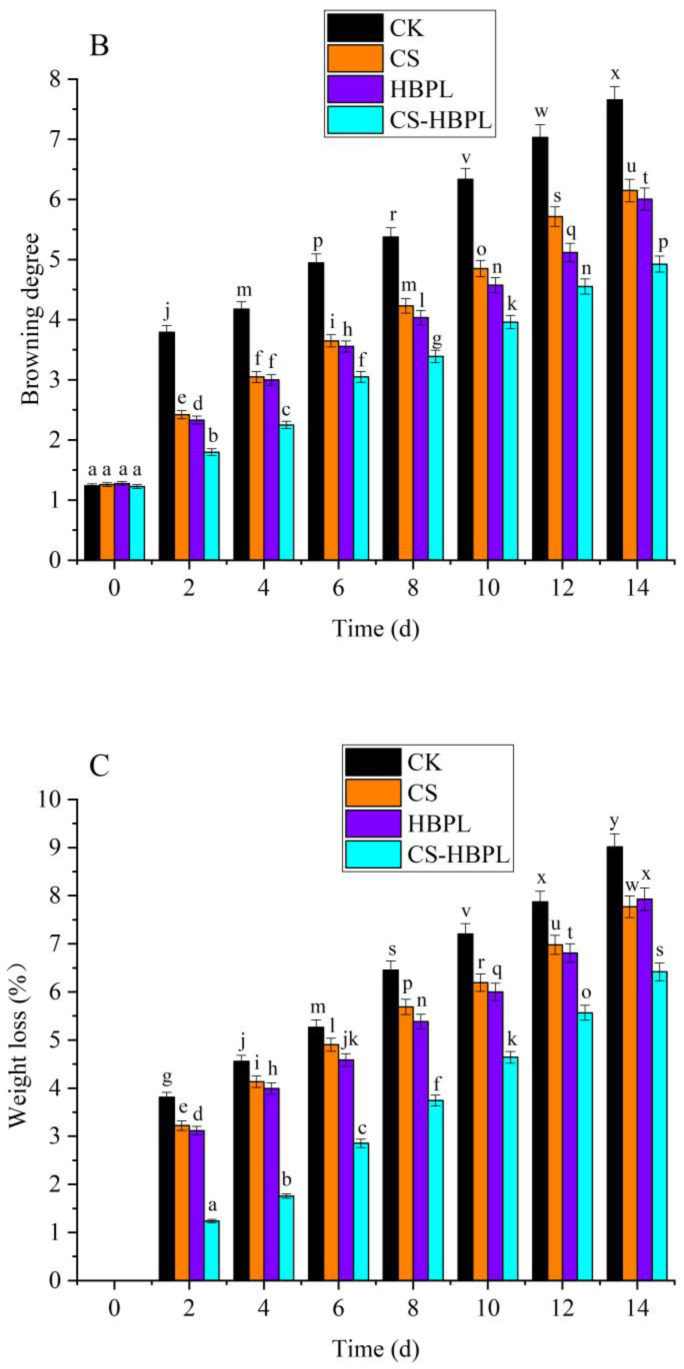
Effects of different treatments on quality characteristics during oyster mushroom storage. (**A**) Rot index; (**B**) Browning degree; (**C**) Weight loss. Vertical bars indicate standard deviation. Values with different letters denote the significant differences (*p* < 0.05).

The rot index of oyster mushroom increased with the extension of storage time (Figure 1A). At 2 d storage, there was no rot phenomenon observed in the treatment and CK groups. However, after 4 d storage, oyster mushroom of both the treatment and control groups began to rot; the rot degree of oyster mushroom in the treatment groups was significantly lower than in the CK group (*p* < 0.05), and the rot degree of the CS-HBPL group was significantly lower than in other treatment groups (*p* < 0.05). At 6 d, the rot degree of the CK group was higher than 0.5, while the rot degree of the treatment group was still lower than 0.4, and that of the CS-HBPL group was the lowest. At 12 d, the rot degree of the CK group was higher than 0.8, while the rot degree of the treatment groups was close to 0.7, and that of the CS-HBPL group was only 0.48. At 14 d, the rot degree of the CK group was more than 0.9; the rot degree of oyster mushroom in the treatment groups was significantly lower than in the CK group (*p* < 0.05), and the rot degree of the CS-HBPL group was significantly lower than that of other treatment groups (*p* < 0.05). Compared with the CK group, all treatment groups significantly inhibited the rot of oyster mushroom (*p* < 0.05), especially the CS-HBPL group.

The tissues of oyster mushroom still carry on active metabolic activities after harvesting, while the oxidation–reduction imbalance and accumulation of oxidation products in the tissue could lead to browning with the extension of storage time. The browning degree of oyster mushroom increased with the extension of storage time (Figure 1B). After 2 d storage, the browning degree of oyster mushroom in treatment groups was significantly lower than in the CK group (*p* < 0.05), and the browning degree of the CS-HBPL group was significantly lower than that of other treatment groups (*p* < 0.05). At 8 d, the browning degree of oyster mushroom in the CK group was higher than 5, while the browning degree of treatment groups was significantly lower than that of the CK group (*p* < 0.05), and the browning degree of the CS-HBPL group was significantly lower than that of other treatment groups (*p* < 0.05). At 14 d, the browning degree of the CK group was higher than 7, and the browning degree of treatment groups was significantly lower than that of the CK group (*p* < 0.05), and the browning degree of the CS-HBPL group was only 4.92. Compared with the CK group, all treatment groups significantly inhibited the browning of oyster mushroom (*p* < 0.05), especially the CS-HBPL group. The main reason for the browning of edible mushrooms was the effect of PPO and microorganisms on its tissues. The CS-HBPL coating might reduce the number of putrid bacteria, which were responsible for the oxidation of phenol into brown-black melanins, thereby hindering the formation of brown spots and enhancing sensory properties.

Weight loss is mainly relevant to respiration and water transpiration, which is one of the main factors determining the storage quality of oyster mushroom [21]. The weight loss rate of oyster mushroom increased with the extension of storage time (Figure 1C). After 2 d storage, the weight loss rate of oyster mushroom in the treatment groups was significantly lower than in the CK group (*p* < 0.05), and the weight loss rate of the CS-HBPL group was significantly lower than that of other treatment groups (*p* < 0.05). At 14 d, the weight loss rate of the CK group was 9.02%, and the weight loss rate of the treatment groups was significantly lower than that of the CK group (*p* < 0.05), with the CS-HBPL group only 6.42%. Compared with the CK group, all treatment groups could significantly reduce the mass loss of oyster mushroom (*p* < 0.05), especially the CS-HBPL group. The loss of water could lead to the shriveling, wilting, and cracking of the fruiting body, which not only severely affects the sensory quality of edible fungi but also alters the freshness, flavor, and weight. Additionally, mass loss could cause an increase in enzyme activity such as PPO in the mushroom, leading to browning. The results were consistent with those published by Liu et al. [20], who reported that the application of *Oudemansiella radicata* polysaccharide coating could reduce the mass loss of oyster mushroom stored at 4 °C. The lower weight loss rate of oyster mushroom in the treatment groups might be due to the semi-permeable barrier capability of CS and HBPL coatings, which could prevent the movement of O_2_, CO_2_, water, and solutes, thereby reducing the rate of respiration, water loss, and oxidation reactions [20].

### 3.2. Effects of CS and HBPL Treatment on Reducing Sugar, Soluble Protein, Total Phenol, and Vc Content during Oyster Mushroom Storage

**Figure 2:**
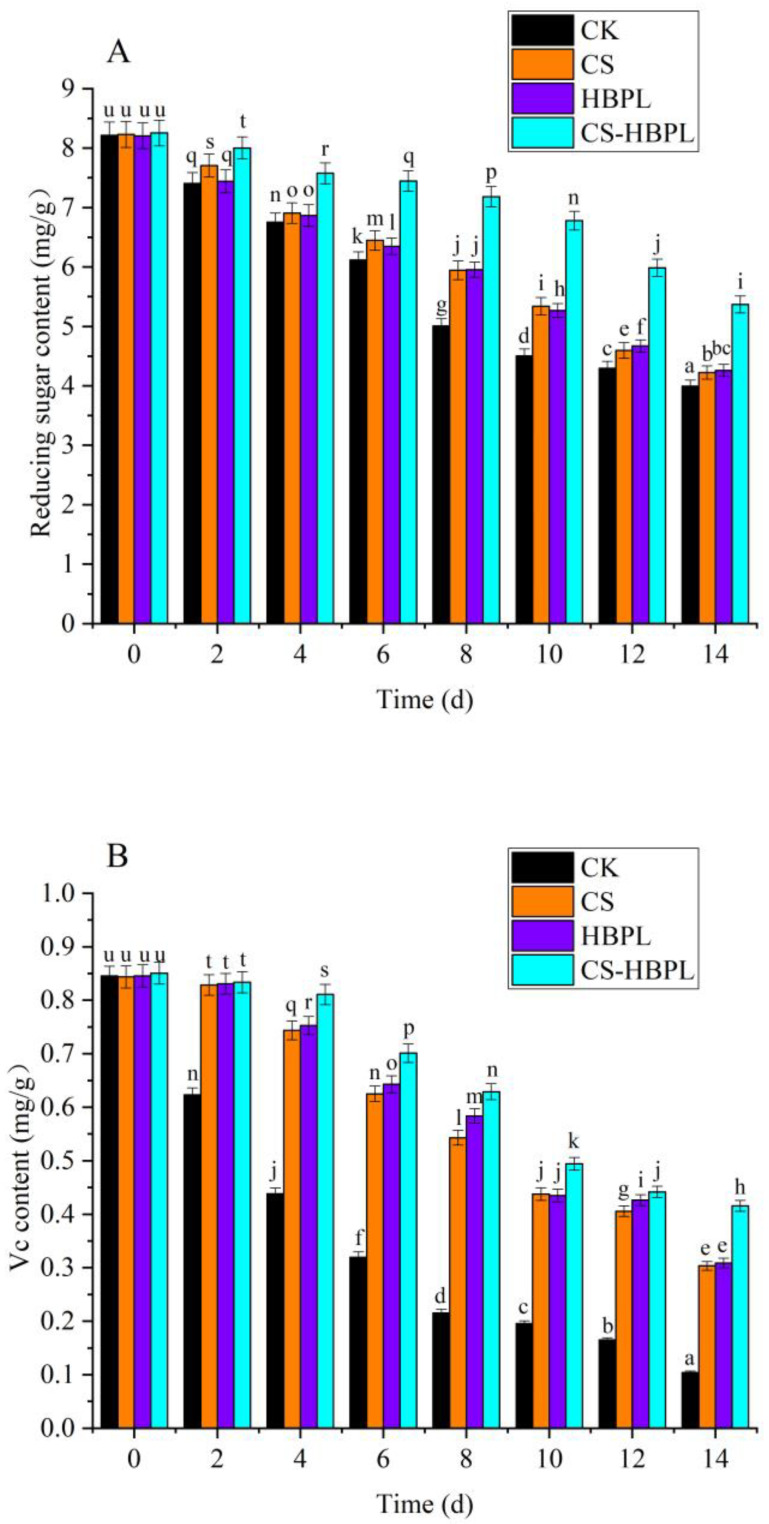

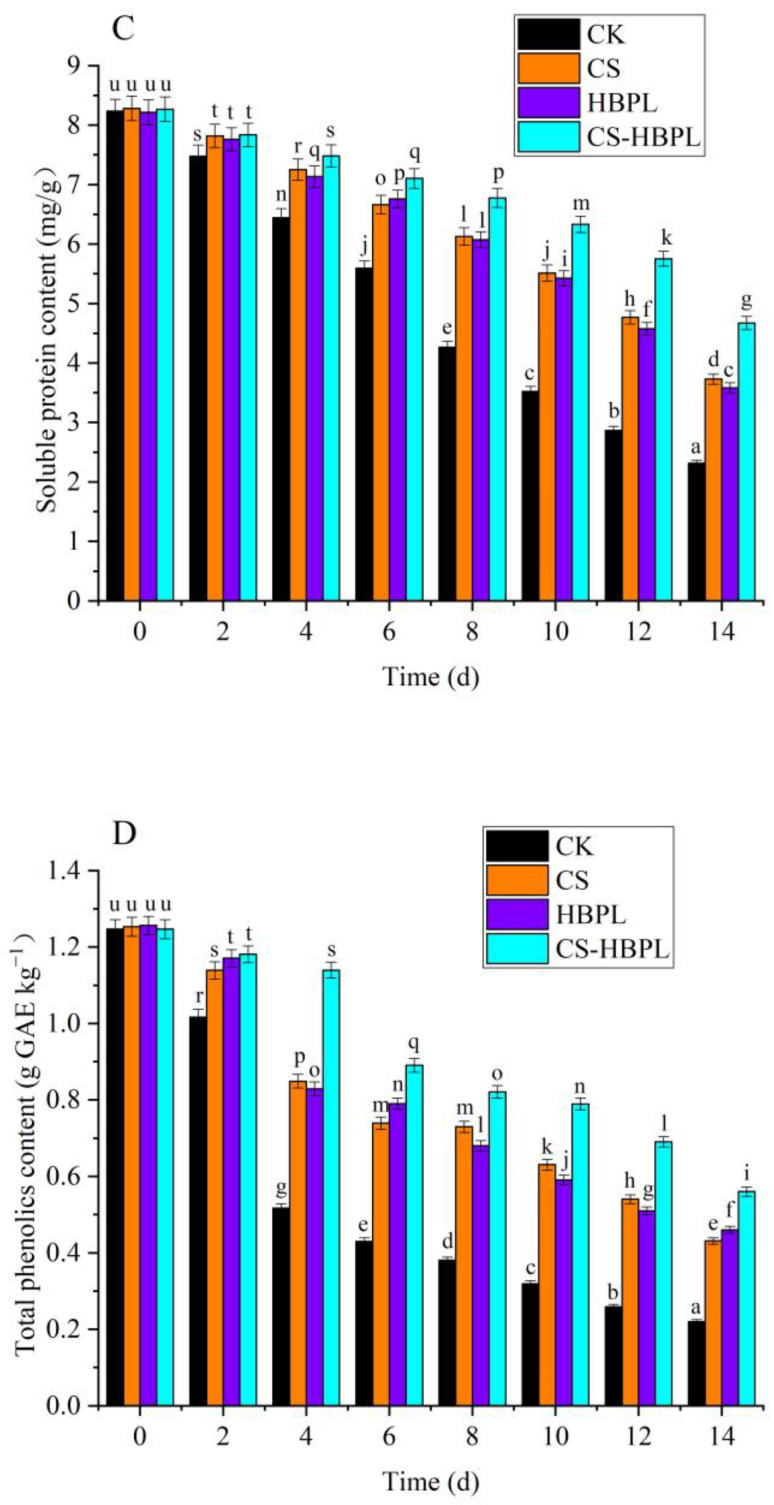
Effects of different treatments on reducing sugar, Vc, soluble protein, and total phenol content during oyster mushroom storage. (**A**) Reducing sugar; (**B**) Vc; (**C**) Soluble protein; (**D**) total phenol. Vertical bars indicate standard deviation. Values with different letters denote the significant differences (*p* < 0.05).

Reducing sugar was an important respiratory substrate for postharvest oyster mushroom, and its content gradually decreased with storage time. As shown in Figure 2A, the reducing sugar content of oyster mushroom gradually decreased with the extension of storage time. At 2 d storage, the reducing sugar content of oyster mushroom in the CS-HBPL and CS groups was significantly higher than that of CK group (*p* < 0.05), and the reducing sugar content of the CS-HBPL group was significantly higher than that of other treatment groups (*p* < 0.05). After 4 d storage, the reducing sugar content of the treatment groups was significantly higher than that of the CK group (*p* < 0.05), and the reducing sugar content of the CS-HBPL group was significantly higher than that of other treatment groups (*p* < 0.05). At 14 d, the reducing sugar content of the CK group was 4.00 mg/g, and the reducing sugar content of the treatment groups was significantly higher than that of the CK group (*p* < 0.05); the reducing sugar content of the CS-HBPL group was 5.37 mg/g, significantly higher than that of the other treatment groups (*p* < 0.05). Compared with the CK group, all treatment groups could significantly reduce the reducing sugar consumption of oyster mushroom (*p* < 0.05), especially the CS-HBPL group.

The antioxidant capacity of oyster mushroom was determined via various compounds, including vitamins, phenols, polysaccharides, and carotenoids [22]. Vitamin C is a strong antioxidant that could prevent or reduce damage caused by reactive oxygen species. The content of Vc not only affects the antioxidant ability of oyster mushroom but also is an important freshness indicator [23]. The Vc content of oyster mushroom gradually decreased with the extension of storage time (Figure 2B). After 2 d storage, the Vc content of oyster mushroom in the treatment groups was significantly higher than that of the CK group (*p* < 0.05). After 4 d storage, the Vc content of the CS-HBPL group was significantly higher than that of the CK group and other treatment groups (*p* < 0.05). At 14 d, the Vc content of the CK group was only 0.10 mg/g, and the Vc content of the treatment groups was significantly higher than that of CK group (*p* < 0.05); the Vc content of the CS-HBPL group was 0.42 mg/100 g, significantly higher than that of other treatment groups (*p* < 0.05). Compared with the CK group, all treatment groups could significantly reduce the Vc consumption of oyster mushroom (*p* < 0.05), especially the CS-HBPL group. The presence of O_2_ would accelerate the loss of ascorbic acid in oyster mushroom, while the CS-HBPL coating slowed down the diffusion of O_2_ and reduced the respiration rate, delaying the ripening speed of oyster mushroom and better maintaining the ascorbic acid content. This work was in good agreement with that reported by Liu et al. [20], who reported that *Oudemansiella radicata* polysaccharide-coated samples (oyster mushroom) had higher ascorbic acid contents.

As the main nutrient of postharvest edible mushrooms, soluble protein is constantly consumed and transformed to satisfy their metabolic activities, and a decrease in soluble protein content is considered to be an important sign of edible fungi tissue aging [20]. The soluble protein content of oyster mushroom gradually decreased with the extension of storage time (Figure 2C). After 2 d storage, the soluble protein content of oyster mushroom in the treatment groups was significantly higher than in the CK group (*p* < 0.05). After 4 d storage, the soluble protein content of the CS-HBPL group was significantly higher than that of the CK group and other treatment groups (*p* < 0.05). At 14 d, the soluble protein content of the CK group was only 2.31 mg/g, and the soluble protein content of the treatment groups was significantly higher than that of the CK group (*p* < 0.05); the soluble protein content of the CS-HBPL group was 4.67 mg/g, significantly higher than that of other treatment groups (*p* < 0.05). Compared with the CK group, all treatment groups could significantly reduce the soluble protein consumption of oyster mushroom (*p* < 0.05), especially the CS-HBPL group. This might be due to the CS-HBPL coating, which could slow down the respiratory rate of oyster mushroom, delay the decomposition of proteins and carbohydrates, and hinder postharvest aging.

As important metabolites in edible fungi tissues, phenols have various physiological functions such as antioxidant, immune enhancement, and antibacterial activities, and they are closely related to the stress resistance, nutritional properties, and sensory quality of postharvest fruiting bodies [24]. The change in phenols content is the main factor affecting the color and browning of postharvest edible mushrooms, and phenols content is closely related to browning. The oxidation of phenols seriously affected the appearance quality and commercial value of edible mushrooms after harvest [25]. The total phenolic content of oyster mushroom gradually decreased with the extension of storage time (Figure 2D). After 2 d storage, the total phenolic content of oyster mushroom in the treatment groups was significantly higher than in the CK group (*p* < 0.05). After 4 d storage, the total phenolic content of the CS-HBPL group was significantly higher than that of the CK group and other treatment groups (*p* < 0.05). At 14 d, the total phenolic content of the CK group was only 0.22 g GAE kg^−1^, and the total phenolic content of the treatment groups was significantly higher than that of the CK group (*p* < 0.05); the total phenolic content of the CS-HBPL group was 0.56 g GAE kg^−1^, significantly higher than that of other treatment groups (*p* < 0.05). Compared with the CK group, all treatment groups could significantly reduce the total phenolic loss of oyster mushroom (*p* < 0.05), especially the CS-HBPL group.

### 3.3. Effects of CS and HBPL Treatment on MDA and Relative Electrolyte Leakage Rate during Oyster Mushroom Storage

**Figure 3:**
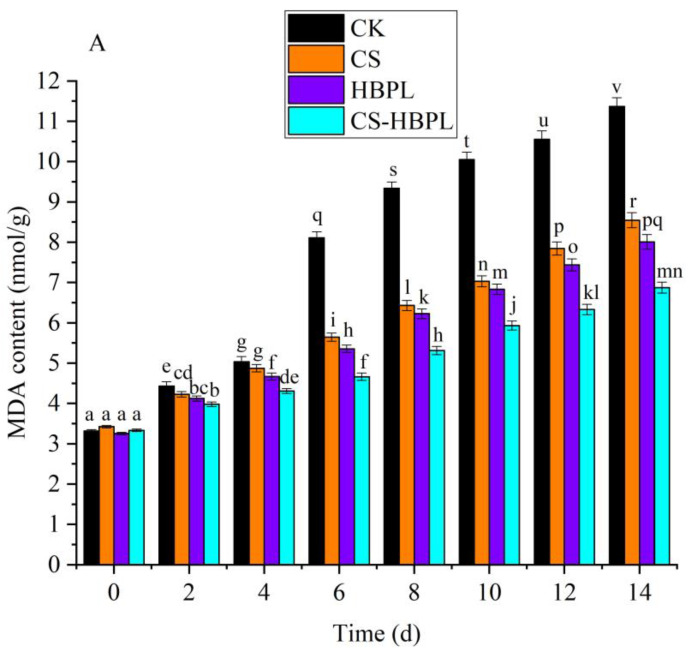

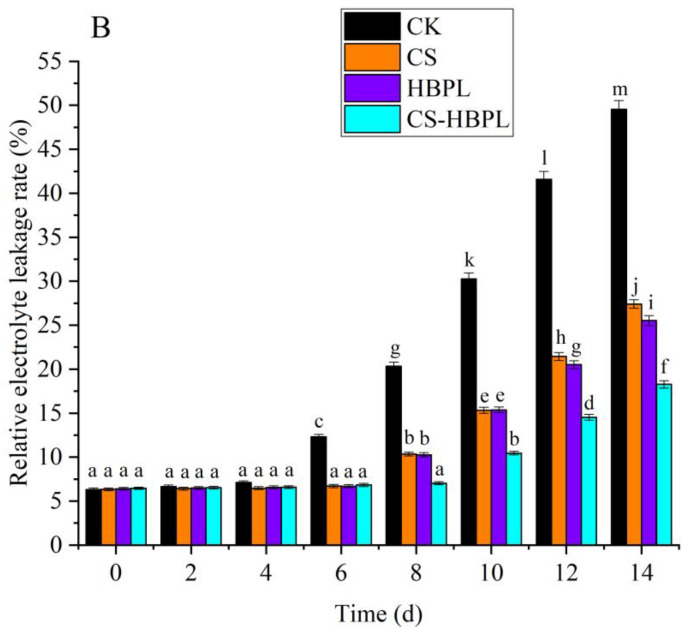
Effects of different treatments on MDA and relative electrolyte leakage rate during oyster mushroom storage. (**A**) MDA; (**B**) Relative electrolyte leakage. Vertical bars indicate standard deviation. Values with different letters denote the significant differences (*p* < 0.05).

Membrane lipid peroxidation often occurs when plant organs are aged or damaged under stress conditions. MDA is the final product of membrane lipid peroxidation, which is an important sign of membrane system damage. A reduction in membrane integrity is one consequence of membrane damage, leading to increased membrane leakage and enhanced cell aging [26]. The MDA content of oyster mushroom gradually increased with the extension of storage time (Figure 3A). After 2 d storage, the MDA content of oyster mushroom in treatment groups was significantly lower than in the CK group (*p* < 0.05). After 4 d storage, the MDA content of the CS-HBPL group was significantly lower than that of the CK group and other treatment groups (*p* < 0.05). At 14 d, the MDA content of the CK group was 11.37 nmol/g, and the MDA content of the treatment groups was significantly lower than that of the CK group (*p* < 0.05); the MDA content of the CS-HBPL group was 6.87 nmol/g, significantly lower than that of other treatment groups (*p* < 0.05). Compared with the CK group, all treatment groups could significantly inhibit an increase in MDA (*p* < 0.05), especially the CS-HBPL group.

Another important change in the quality degradation of postharvest edible mushrooms is that the permeability of the biofilm increases after irreversible damage due to the increase in MDA content, while the unsaturation of membrane lipid decreases, resulting in decreased membrane fluidity, the leakage of intracellular electrolyte, and an increase in relative conductivity [20]. Therefore, the relative electrolyte leakage rate could be used to assess the changes in the membrane permeability of edible mushrooms. The relative electrolyte leakage rate of oyster mushroom gradually increased with the extension of storage time (Figure 3B). After 6 d storage, the relative electrolyte leakage rate of oyster mushroom in treatment groups was significantly lower than in the CK group (*p* < 0.05). After 8 d storage, the relative electrolyte leakage rate of the CS-HBPL group was significantly lower than in the CK group and other treatment groups (*p* < 0.05). At 14 d, the relative electrolyte leakage rate of the CK group was 49.54%, and the relative electrolyte leakage rate of the treatment groups was significantly lower than that of the CK group (*p* < 0.05); the relative electrolyte leakage rate of the CS-HBPL group was 18.27%, significantly lower than that of other treatment groups (*p* < 0.05). Compared with the CK group, all treatment groups could significantly reduce the relative electrolyte leakage rate (*p* < 0.05), especially the CS-HBPL group. The results indicated that the CS-HBPL coating had a good protective effect on the damage of membrane permeability and could effectively put off lipid peroxidation and membrane destruction, retard aging, and prolong the shelf life of oyster mushroom. Similar results were obtained by Liu et al. [20], who found that *Oudemansiella radicata* polysaccharide coating retarded the increase in MDA and relative electrolyte leakage in oyster mushroom.

### 3.4. Effects of CS and HBPL Treatment on Enzyme Activity during Oyster Mushroom Storage

CAT, SOD, POD, and PAL are important enzyme indicators for evaluating the stress resistance of edible fungi. ROS are produced during the normal physiological metabolism of plants, and the plant body will initiate a corresponding defense and regulatory system to alleviate the production of ROS. SOD, POD, and CAT have distinct scavenging effects on ROS in the body [5]. SOD can remove the superoxide anion free radicals produced by metabolism in plants and induce them to convert into H_2_O_2_ with lower toxicity. POD and CAT can effectually remove H_2_O_2_ produced by metabolism in plants and decompose it into non-toxic H_2_O and O_2_.

**Figure 4:**
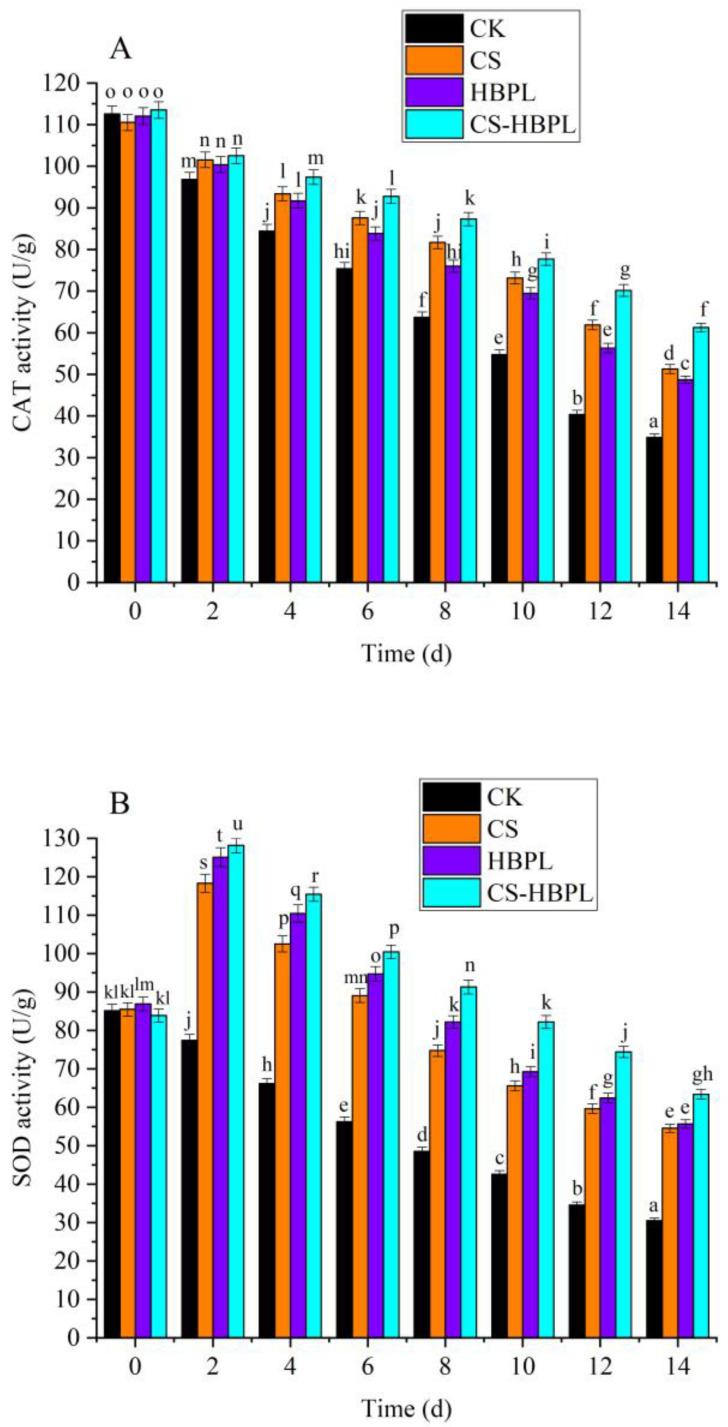

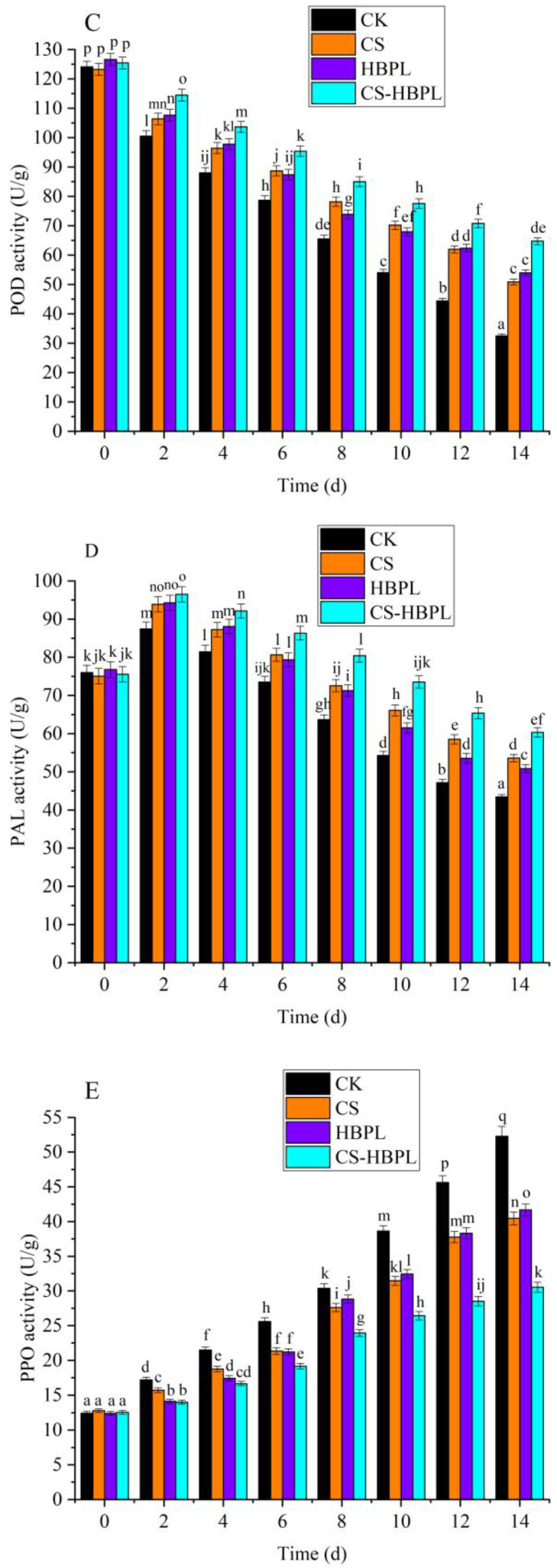
Effects of different treatments on CAT, SOD, POD, PAL, and PPO activities during oyster mushroom storage. (**A**) CAT; (**B**) SOD; (**C**) POD; (**D**) PAL; (**E**) PPO. Vertical bars indicate standard deviation. Values with different letters denote the significant differences (*p* < 0.05).

The CAT activity of oyster mushroom increased throughout the storage period (Figure 4A). After 2 d storage, the CAT activity of oyster mushroom in the treatment groups was significantly higher than in the CK group (*p* < 0.05). After 4 d storage, the CAT activity of the CS-HBPL group was significantly higher than that of the CK group and other treatment groups (*p* < 0.05). At 14 d, the CAT activity of the CK group was 34.84 U/g, and the CAT activity of the treatment groups was significantly higher than that of the CK group (*p* < 0.05); the CAT activity of the CS-HBPL group was 61.24 U/g, significantly higher than that of other treatment groups (*p* < 0.05). Compared with the CK group, all treatment groups significantly increased the CAT activity (*p* < 0.05), especially the CS-HBPL group. The increase in CAT activity could remove H_2_O_2_, reduce the oxidative damage of free radicals, and inhibit the browning of oyster mushroom.

As shown in Figure 4B, the SOD activity of oyster mushroom in the control group decreased during storage, while the SOD activity of the treatment groups first increased and then decreased during storage. After 2 d storage, the SOD activity of oyster mushroom in the treatment groups was significantly higher than in the CK group (*p* < 0.05), and the SOD activity of the CS-HBPL group was significantly higher than that of other treatment groups (*p* < 0.05). At 14 d, the SOD activity of the CK group was 30.51 U/g, and the SOD activity of the treatment groups was significantly higher than that of the CK group (*p* < 0.05); the SOD activity of the CS-HBPL group was 63.39 U/g, significantly higher than that of other treatment groups (*p* < 0.05). Compared with the CK group, all treatment groups significantly increased the SOD activity of oyster mushroom (*p* < 0.05), especially the CS-HBPL group. During the senescence of plants, SOD activity decreased and reactive oxygen species accumulated such as H_2_O_2_ and O^·2−^, which enhanced the peroxidation of the plasma membrane and led to membrane damage. SOD, as an endogenous reactive oxygen species scavenger, could remove excessive reactive oxygen species in plants during the aging process, maintain the oxygen metabolism balance, and preserve membrane structural integrity, thereby delaying the aging process to a certain extent.

In the presence of H_2_O_2_, POD could effectively decompose lethal and pathogenic oxidized phenolic substances in plants, promoting self-defense against these substances in the organism [5]. As shown in Figure 4C, the POD activity of oyster mushroom decreased throughout the storage period. After 2 d storage, the POD activity of oyster mushroom in the treatment groups was significantly higher than in the CK group (*p* < 0.05), and the POD activity of the CS-HBPL group was significantly higher than that of other treatment groups (*p* < 0.05). At 14 d, the POD activity of the CK group was 32.51 U/g, and the POD activity of the treatment groups was significantly higher than that of the CK group (*p* < 0.05); the POD activity of the CS-HBPL group was 64.73 U/g, significantly higher than that of other treatment groups (*p* < 0.05). Compared with the CK group, the POD activity of all treatment groups was significantly improved (*p* < 0.05), especially the CS-HBPL group. POD was an important terminal oxidase involved in the cell respiratory metabolic process, which helped to eliminate H_2_O_2_ in plants and prevent the body from being poisoned; thus, higher POD activity could delay cell aging to a certain extent [27].

As shown in Figure 4D, the PAL activity of oyster mushroom initially increased and then decreased throughout the storage period. At 2 d storage, the PAL activity of oyster mushroom in the CK group and treatment groups increased, while the PAL activity of treatment groups was significantly higher than that of the CK group (*p* < 0.05). After 4 d storage, the PAL activity of the CK and treatment groups decreased; the PAL activity of the treatment groups was significantly higher than that of the CK group (*p* < 0.05), and the PAL activity of the CS-HBPL group was significantly higher than that of other treatment groups (*p* < 0.05). At 14 d, the PAL activity of the CK group was 43.41 U/g, and the PAL activity of the treatment groups was significantly higher than that of the CK group (*p* < 0.05); the PAL activity of the CS-HBPL group was 60.33 U/g, significantly higher than that of other treatment groups (*p* < 0.05). Similar results were obtained by Guo et al. [5], who found that shiitake mushrooms’ polysaccharide and chitosan coating was able to maintain high PAL activity. The PAL activity gradually increased in the early stage of oyster mushroom storage, which might be a response of the tissue to mechanical damage. Subsequently, the PAL activity significantly decreased, likely due to the oxidative damage of enzyme proteins caused by ROS accumulation in cells [28]. PAL was a key enzyme in the phenylpropanoid pathway, which could catalyze the synthesis of various secondary metabolites (such as phenols, flavonoids, and lignin) and was closely related to the stress and disease resistance of edible fungi, playing an important role in the browning, softening, and aging processes of postharvest edible fungi [29,30].

PPO is one of the main enzymes that causes enzymatic browning in edible fungi. PPO could catalyze the oxidation of various phenols into quinone compounds, which could further produce melanin and cause the browning of edible fungi [31]. In the storage process, an increase in PPO content could accelerate the surface browning of edible fungi, and inhibiting PPO activity could effectively slow down the browning. The PPO activity of oyster mushroom increased throughout the storage period (Figure 4E). After 2 d storage, the PPO activity of oyster mushroom in the treatment groups was significantly lower than that of the CK group (*p* < 0.05). After 6 d storage, the PPO activity of the CS-HBPL group was significantly lower than that of the CK group and other treatment groups (*p* < 0.05). At 14 d, the PPO activity of the CK group was 52.27 U/g, and the PPO activity of the treatment groups was significantly lower than that of the CK group (*p* < 0.05); the PPO activity of the CS-HBPL group was 30.53 U/g, significantly lower than that of other treatment groups (*p* < 0.05). Compared with the CK group, all treatment groups could significantly inhibit PPO activity (*p* < 0.05), especially the CS-HBPL group.

SOD and CAT played important roles in antioxidant defense during plant maturation and aging [32]. The enhancing activity of these antioxidant enzymes was necessary for reducing oxidative damage caused by free radicals, and the CS-HBPL coating helped to maintain high enzyme activity and reduce oxidative stress, thereby extending the shelf life of oyster mushroom. PPO was involved in the oxidation of phenols, leading to the browning of harvested fruits and vegetables [33]. The activity of PPO increased during oyster mushroom storage (Figure 4E), which was well correlated with the browning outcome of oyster mushroom (Figure 1B).

In summary, CS-HBPL coating, as a protective layer on the surface of fresh oyster mushroom, could effectively reduce the oxidation of phenols, delay browning, and inhibit the maturation and aging of oyster mushroom.

### 3.5. Correlation Analysis of All the Indexes during Storage

The correlation analysis of all the indexes is shown in Table 1. Browning was significantly positively correlated with rot degree, weight loss, MDA, relative electrolyte leakage, and PPO activity. Conversely, there was a significant negative correction between browning and reducing sugar, Vc, total phenolic, soluble protein, and CAT, SOD, POD, and PAL activity. It could be reflected that browning is closely related to the relevant enzymes in tissues and the related components.

Browning is a characteristic of fruit ripening and aging, which is an important indicator for evaluating fruit storage quality. Browning is caused by a variety of factors, such as phenolic content, phenolic enzyme activity, reactive oxygen species, and membrane lipid peroxidation [34]. PAL could promote the synthesis of phenols, while PPO could react with phenols, resulting in the browning of mushrooms. Correlation analysis showed that PAL was significantly positively correlated with total phenolic, while PPO was significantly negatively correlated with total phenolic.

A CS-HBPL coating could significantly improve the POD, SOD, and CAT activities of antioxidant enzymes. Correlation analysis showed that browning was significantly negatively correlated with POD, SOD, and CAT activity. Therefore, the CS-HBPL coating could inhibit the browning of oyster mushroom by improving the ability of oyster mushroom to scavenge ROS and reducing the oxidative damage caused by free radicals.

## 4. Conclusions

In summary, CS-HBPL combined treatment could significantly reduce rot degree and weight loss and significantly inhibit the browning of oyster mushroom; the loss of reducing sugar, Vc, soluble protein, and phenols in oyster mushroom was significantly reduced. Compared with the control, CS-HBPL combined treatment could significantly inhibit an increase in MDA and significantly decrease the relative electrolyte leakage of oyster mushroom. In addition, the activities of CAT, SOD, PAL, and POD were significantly increased, and the activity of PPO was significantly inhibited. With the extension of storage time, the texture, odor, and folds color of oyster mushroom changed to different degrees. After 6 d storage, the oyster mushroom of the CK group showed obvious browning, odor, and stickiness, while the oyster mushroom of the CS-HBPL group showed basically no browning, odor, or stickiness. After 12 d storage, the oyster mushroom of the CK group showed severe browning and odor, and the mushroom body was obviously sticky and soft; however, the oyster mushroom of the CS-HBPL group showed slight browning, odor, and stickiness. Compared with the CK group, the oyster mushroom of the CS-HBPL group still showed better sensory quality after storage for 12 days. In conclusion, CS-HBPL combined treatment could effectively maintain the quality characteristics and nutritional qualities of oyster mushroom, provide protective effects on membrane permeability damage, effectively delay the oxidation of phenols and browning, and inhibit the maturation and aging of oyster mushroom, extending the shelf life. Although in-depth studies are needed to completely elucidate the mechanism of combined CS and HBPL-based coating to extend the shelf life of oyster mushroom, we could speculate that HBPL could be used as a potential food preservative.

## Figures and Tables

**Table 1:** Pearson correlation analysis of all the indexes during storage.

	RD	BR	WL	RS	Vc	TP	SP	MDA	REL	CAT	SOD	POD	PAL	PPO
RD	1	0.947 **	0.930 **	−0.972 **	−0.941 **	−0.954 **	−0.960 **	0.939 **	0.821 **	−0.956 **	−0.840 **	−0.955 **	−0.869 **	0.949 **
BR		1	0.969 **	−0.959 **	−0.967 **	−0.961 **	−0.972 **	0.960 **	0.865 **	−0.975 **	−0.828 **	−0.979 **	−0.811 **	0.956 **
WL			1	−0.962 **	−0.907 **	−0.940 **	−0.930 **	0.904 **	0.772 **	−0.955 **	−0.719 **	−0.974 **	−0.742 **	0.924 **
RS				1	0.920 **	0.932 **	0.969 **	−0.934 **	−0.841 **	0.973 **	0.797 **	0.967 **	0.860 **	−0.962 **
Vc					1	0.964 **	0.958 **	−0.958 **	−0.842 **	0.943 **	0.899 **	0.939 **	0.838 **	−0.918 **
TP						1	0.939 **	−0.933 **	−0.778 **	0.941 **	0.823 **	0.949 **	0.776 **	−0.902 **
SP							1	−0.972 **	−0.920 **	0.987 **	0.847 **	0.967 **	0.883 **	−0.975 **
MDA								1	0.911 **	−0.948 **	−0.841 **	−0.940 **	−0.835 **	0.941 **
REL									1	−0.883 **	−0.804 **	−0.839 **	−0.864 **	0.915 **
CAT										1	0.800 **	0.986 **	0.856 **	−0.978 **
SOD											1	0.776 **	0.911 **	−0.824 **
POD												1	0.816 **	−0.967 **
PAL													1	−0.897 **
PPO														1

Note: RD, BR, WL, RS, TP, SP, and REL meant rot degree, browning, weight loss, reducing sugar, total phenolic, soluble protein, and relative electrolyte leakage, respectively. ** The difference was significant at the 0.01 level.

## Data Availability

The data presented in this study are available on request from the corresponding author. Relevant information and techniques have been provided in this study.
